# Invasive tracheal mucormycosis causing irreversible glottic closure and long-term tracheostomy dependence: a case report and airway-centered management implications

**DOI:** 10.3389/fmed.2026.1859562

**Published:** 2026-06-16

**Authors:** Yuanjiang Zheng, Hengzhi Chen, Yucai Li, Fei Luo, Xianwei Ye

**Affiliations:** 1 Department of Respiratory and Critical Care Medicine, Xingyi People's Hospital, Xingyi, Guizhou, China; 2 Department of Respiratory and Critical Care Medicine, Guizhou Provincial People's Hospital, Guiyang, Guizhou, China; 3 Department of Medical Imaging, Xingyi People's Hospital, Xingyi, Guizhou, China

**Keywords:** airway management, antifungal therapy, diabetes, invasive mucormycosis, tracheal infection, tracheostomy

## Abstract

Invasive tracheal mucormycosis is a rare but rapidly progressive central airway fungal infection associated with high mortality. Its early clinical manifestations and radiological features are often nonspecific, which may result in delayed diagnosis. We report a case of invasive tracheal mucormycosis in a patient with diabetic ketosis. The patient underwent emergency tracheostomy for necrotizing upper airway obstruction. Bronchoscopic examination revealed extensive white necrotic tissue in the subglottic region and trachea. Broad, ribbon-like, pauci-septate hyphae were identified in both bronchoalveolar lavage fluid and biopsy specimens, establishing the diagnosis. Following systemic antifungal therapy with amphotericin B, adjunctive nebulized local treatment, and repeated bronchoscopic interventions, the infection was controlled, and maintenance therapy was sequentially transitioned to oral isavuconazole. However, the patient ultimately developed irreversible glottic closure and severe subglottic stenosis, resulting in long-term tracheostomy dependence. This case highlights that mucormycosis should be considered early in the differential diagnosis of patients with diabetes, particularly those with diabetic ketosis, who present with necrotizing or pseudomembranous airway lesions on bronchoscopy and non response to broad spectrum antibiotics. Deep respiratory specimens should be obtained promptly to establish etiological and histopathological diagnoses, while adequate antifungal therapy and correction of metabolic disturbances should be initiated simultaneously to improve outcomes. Nevertheless, even after infection control is achieved, patients may still be left with severe structural airway damage. Therefore, airway function preservation and long-term prognostic assessment should also be emphasized in clinical management.

## 1 Introduction

Mucormycosis is an invasive fungal infection caused by fungi of the order Mucorales. Although uncommon overall, it is characterized by marked tissue invasiveness, rapid progression, and poor prognosis (1, 2). Systematic reviews and landmark reviews have shown that diabetes is one of the most common underlying conditions, with the rhino-orbital-cerebral form being the predominant clinical presentation, followed by pulmonary involvement (3, 4). In contrast, cases primarily involving the central airway, including the larynx, subglottic region, or trachea, have thus far been reported mainly in isolated case reports and small case series, suggesting that this represents a much rarer clinical phenotype from an epidemiological perspective (5, 6). In such uncommon presentations, early manifestations are often limited to fever, cough, dyspnea, wheezing, hoarseness, or progressive upper airway obstruction. Both clinical and radiological findings are usually nonspecific, and the disease can therefore be easily mistaken for common respiratory tract infection, airway malignancy, or other central airway disorders, resulting in delayed diagnosis (5–8).

Invasive tracheal mucormycosis (ITM) warrants particular attention because the site of involvement directly affects airway patency. Once necrotizing or pseudomembranous obstruction develops, the condition may rapidly progress to life-threatening central airway obstruction (5, 6, 8). Existing literature has focused largely on infection control and short-term survival, whereas reports describing residual laryngotracheal scarring, fixed stenosis, and long-term dependence on an artificial airway after infection remission remain limited (5, 8). Here, we report a case of ITM in a patient with diabetic ketosis, with the aim of improving clinical awareness of the early recognition, timely diagnosis, and long-term airway outcomes of central airway mucormycosis.

## 2 Case presentation

A 51-year-old woman was admitted to the emergency department of our hospital on February 3, 2024, with a 20-day history of cough, purulent sputum, and fever, along with a 9-day history of dyspnea. Twenty days before admission, she developed cough with yellow purulent sputum and a peak temperature of 39.2 °C. During anti-infective treatment at a local clinic, her dyspnea gradually worsened, and arterial blood gas analysis indicated respiratory failure. She was subsequently transferred to the intensive care unit of a municipal hospital. Bronchoscopic examination revealed a suspected fistula in the upper trachea and inflammatory changes in the bronchial mucosa. Marked dyspnea occurred during the procedure, and emergency tracheostomy with endoscopic intervention was performed. Bronchoscopy showed that the lumen of the upper trachea was obstructed by a large amount of yellow-white necrotic tissue, which could not be completely removed despite repeated forceps extraction and cryotherapy. Although empirical anti-infective therapy and other supportive measures were administered, the patient remained febrile and was therefore transferred to our hospital for further evaluation and treatment.

**Figure 1:**
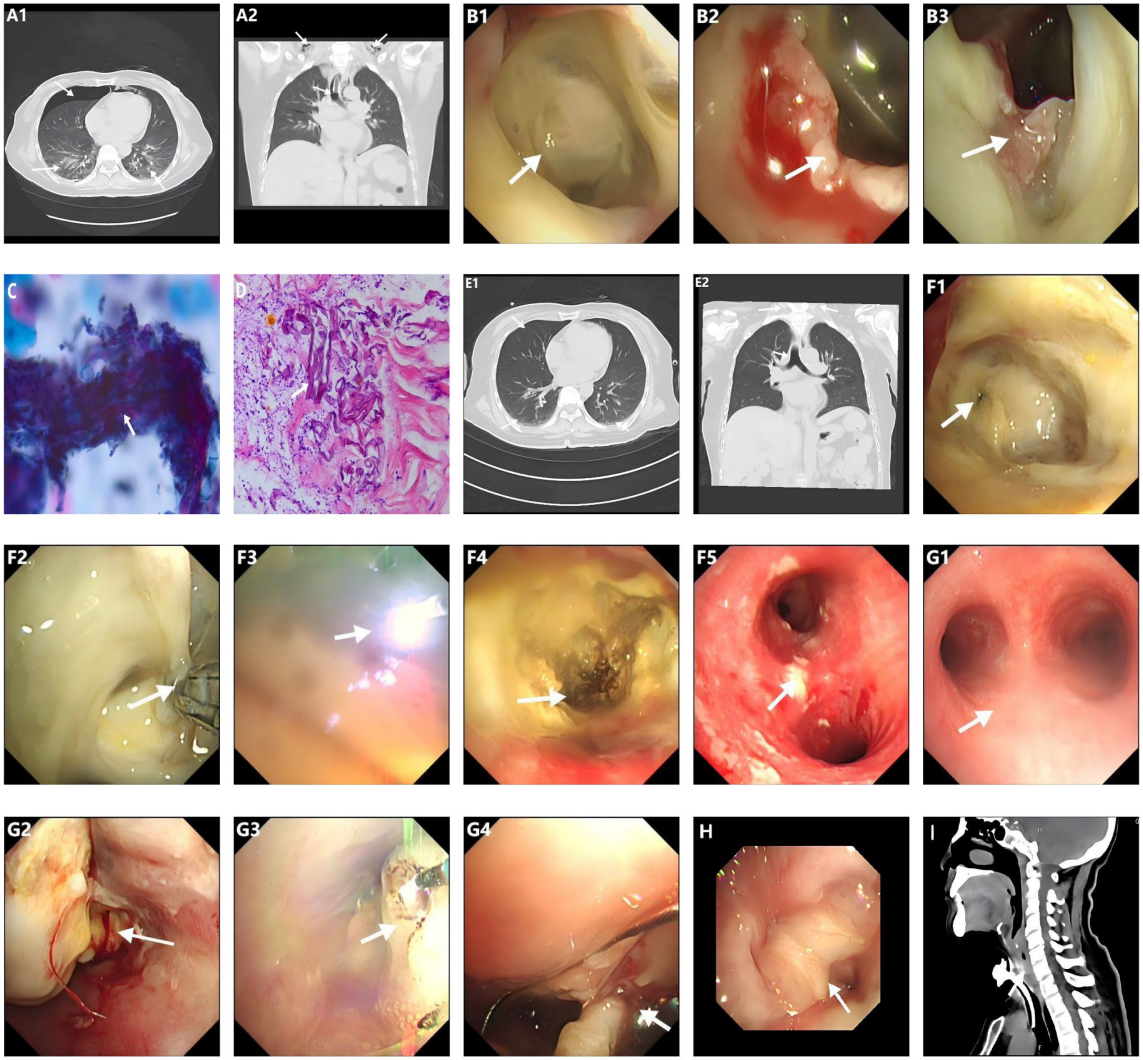
**(A)** CT performed on February 3, 2024, showing multiple bilateral inflammatory infiltrates and exudative lesions, predominantly involving the lower lobes. A right-sided pneumothorax is present **(A1)**. Scattered subcutaneous and mediastinal emphysema is observed in the bilateral thoracic inlet, anterior upper chest wall, and mediastinum **(A2)**. **(B)** Fiberoptic bronchoscopy performed on February 4, 2024, showing extensive white necrotic debris below the glottis causing marked airway narrowing **(B1)**, granulation tissue proliferation around the tracheostomy tube **(B2)**, and abundant whitish viscid secretions in the lower trachea **(B3)**. **(C)** Microbiological examination of bronchoalveolar lavage fluid obtained on February 5, 2024, revealing broad, ribbon-like, pauci-septate fungal hyphae morphologically consistent with fungi of the order Mucorales. **(D)** Histopathological examination of biopsy specimens obtained on February 5, 2024, demonstrating extensive necrotic inflammatory tissue containing broad, ribbon-like, pauci-septate fungal hyphae, morphologically consistent with mucormycosis. Special stains were positive for PAS and GMS, whereas acid-fast staining was negative. **(E)** Follow-up chest CT performed on February 8, 2024, showing near-complete resolution of the right-sided pneumothorax and marked absorption of bilateral inflammatory infiltrates **(E1)**; mediastinal and subcutaneous emphysema were also significantly reduced **(E2)**. **(F)** Repeat bronchoscopy performed on February 9, 2024, showing persistent white necrotic debris below the glottis and around the tracheostomy site **(F1)**. The necrotic tissue was firm and difficult to remove using forceps **(F2)**; therefore, laser ablation was performed for bronchoscopic debridement **(F3)**, resulting in enlargement of the subglottic airway lumen **(F4)**. White mucosal plaques in the lower trachea were reduced compared with previous findings **(F5)**. **(G)** Third bronchoscopy performed on February 18, 2024, showing near-complete resolution of the white mucosal plaques in the lower trachea **(G1)**, but persistent white necrotic tissue in the upper trachea with luminal narrowing and mucosal hyperemia **(G2)**. Partial removal and release of the necrotic tissue were attempted using laser and electrosnare techniques **(G3)**, although complete clearance was not achieved **(G4)**. **(H)** Fiberoptic bronchoscopy performed during the second hospitalization on March 19, 2024, demonstrating glottic closure. **(I)** Contrast-enhanced neck CT performed during the third hospitalization on September 3, 2024, showing extensive adhesions involving the glottic and subglottic regions.

The patient had a history of type 2 diabetes mellitus and had been receiving long-term insulin therapy, although glycemic control was poor. On admission, tracheostomy was in place. Subcutaneous crepitus was palpable in the neck and bilateral supraclavicular fossae. Breath sounds over the right lung were decreased, and a few fine moist rales were heard at both lower lung fields. Laboratory tests showed a white blood cell count of 15.70 × 10^9^/L, high-sensitivity C-reactive protein of 19.18 mg/L, random blood glucose of 22.19 mmol/L, and beta-hydroxybutyrate of 4.36 mmol/L. Arterial blood gas analysis showed no respiratory or acidosis at that time. Chest computed tomography (CT) revealed a right pneumothorax with approximately 30–40% lung compression, as well as multiple inflammatory infiltrative and exudative lesions in both lungs, predominantly involving the bilateral lower lobes (Figure 1A1). Scattered gas accumulation was also seen in the bilateral thoracic inlet, anterior upper chest wall, and mediastinum (Figure 1A2). The initial diagnoses were severe bilateral pneumonia, right pneumothorax, suspected tracheal fistula, type 2 diabetes mellitus with ketosis, and status post tracheostomy. After admission, piperacillin-tazobactam was initiated, continuous oxygen was delivered through the tracheostomy, intensive insulin therapy was administered to control hyperglycemia and correct ketosis, and vital signs together with laboratory parameters were closely monitored.

Bronchoscopy performed on February 4, 2024, showed a large amount of white necrotic material in the subglottic area, causing marked luminal narrowing (Figure 1B1); granulation tissue hyperplasia around the tracheostomy tube (Figure 1B1); abundant white, thick secretions in the lower trachea (Figure 1B3); and scattered white plaques on the mucosa of the middle-to-lower trachea, the openings of both main bronchi, and the right middle and lower lobe bronchi. Concurrent microbiological examination of bronchoalveolar lavage fluid identified fungal hyphae with morphological features consistent with Mucorales (Figure 1C). Subsequent biopsy pathology demonstrated broad, ribbon-like, pauci-septate hyphae within extensively inflamed and necrotic tissue, supporting a diagnosis of mucormycosis (Figure 1D). Special stains were positive for periodic acid-Schiff (PAS) and Grocott methenamine silver (GMS), whereas acid-fast staining was negative. Based on the clinical course and imaging findings, a final diagnosis of ITM was established. Intensified antifungal therapy was initiated immediately with amphotericin B cholesterol sulfate complex as the main agent. The patient weighed 65 kg at the initiation of treatment. A stepwise dose-escalation regimen was used, increasing from 50 mg/day, equivalent to 0.77 mg/kg/day, to 250 mg/day, equivalent to 3.85 mg/kg/day. Nebulized amphotericin B at 5 mg twice daily was administered concurrently to enhance local drug exposure.

The right pneumothorax was managed conservatively with oxygen therapy, close clinical monitoring, and supportive treatment, without the need for chest tube drainage. Repeat chest CT on February 8, 2024, showed near-complete resolution of the right pneumothorax and absorption of the bilateral inflammatory exudative lesions (Figure 1E1). Mediastinal and subcutaneous emphysema had also markedly decreased (Figure 1E2). Gastroscopy did not reveal a tracheoesophageal fistula. Repeat bronchoscopy on February 9 showed persistent abundant white necrotic material in the subglottic region. Although the airway was not completely occluded, conspicuous white necrotic tissue remained around the tracheostomy site (Figure 1F1). Removal with biopsy forceps was attempted, but the tissue was firm and difficult to extract (Figure 1F2). Laser ablation was therefore performed to enlarge the lumen (Figures 1F3,F4), and the white plaques in the lower tracheal mucosa were reduced compared with previous findings (Figure 1F5). Following treatment, body temperature returned to normal, and both cough and dyspnea improved significantly. Pulmonary rales disappeared or were markedly alleviated. Peripheral oxygen saturation remained between 96 and 100% under low-flow oxygen therapy, and inflammatory markers together with the white blood cell count gradually declined in parallel with radiological improvement. After stabilization, the patient was transferred to the general ward on February 12 for continued antifungal therapy and respiratory rehabilitation. A third bronchoscopy on February 18, 2024, showed near-complete resolution of the white plaques in the lower tracheal mucosa (Figure 1G1), mild glottic edema, and persistent white necrotic material in the upper trachea causing luminal narrowing, with mucosal congestion (Figure 1G2). Part of the necrotic tissue was removed bronchoscopically, and adhesiolysis was attempted using laser and an electrosurgical snare (Figure 1G3); however, complete removal was not achieved because of the hardness of the necrotic tissue (Figure 1G4). Thereafter, the patient’s symptoms continued to improve. She was discharged on February 22 after marked symptomatic relief and was prescribed oral isavuconazole sulfate for maintenance therapy at 200 mg three times daily for the first 48 h, followed by 200 mg once daily thereafter.

From March 18 to 22, 2024, the patient was readmitted for a second hospitalization to assess the feasibility of decannulation. Her temperature was normal, the tracheostomy remained in place, and phonation was difficult. Bronchoscopy showed glottic closure (Figure 1H). Thickening and narrowing of the opening of the right lower lobe bronchus were also noted. Imaging showed no acute progression compared with previous examinations, but decannulation was considered difficult. Oral isavuconazole at 200 mg once daily, nebulized therapy, standardized tracheostomy care, and metabolic management were continued. After replacement with a metal tracheostomy tube, the patient remained stable and was discharged. During outpatient follow-up through June 22, 2024, imaging showed near-complete absorption of gas in the thoracic inlet and mediastinum, with marked improvement in bilateral pulmonary inflammatory exudation. Isavuconazole was discontinued after comprehensive consideration of the disease status and treatment adherence. From August 31 to September 6, 2024, the patient was hospitalized for a third time for reassessment of decannulation feasibility. Inflammatory markers were normal, and sputum culture was negative. Contrast-enhanced neck CT showed severe glottic and subglottic stenosis with extensive adhesions (Figure 1I). Evaluation by the otolaryngology team concluded that decannulation and closure of the tracheostomy stoma were unlikely to be achieved in the short term. The interventional pulmonology team recommended a staged reconstructive approach, including balloon dilation, scar release, and placement of either a silicone stent or a Montgomery T-tube. Because this strategy would require multiple procedures and carried risks of restenosis and infection, the patient ultimately chose conservative management and was discharged after continued tracheostomy care and rehabilitation training. At telephone follow-up on December 1, 2025, the tracheostomy tube remained in place. The patient still had difficulty speaking and reported occasional nausea and vomiting, but her vital signs were stable.

## 3 Discussion

This case suggests that in ITM, diabetes, particularly when complicated by DKA, is not only a common underlying condition but may also be a major host factor driving rapid disease progression and the development of a necrotizing airway phenotype (6, 9, 10). Previous studies have shown that hyperglycemia, acidosis, and disordered iron metabolism can synergistically impair host defenses while enhancing the adhesion and invasive capacity of Mucorales to host tissues. These mechanisms may promote a localized pattern of progression within the central airway characterized by tissue necrosis, pseudomembrane formation, and luminal obstruction (9, 10). Compared with pulmonary parenchymal mucormycosis, ITM usually lacks distinctive radiologic features. In the early stage, chest CT often shows only airway wall thickening, luminal narrowing, post-obstructive inflammatory changes, or other nonspecific associated abnormalities, making timely diagnosis difficult when imaging findings are interpreted in isolation (5, 6). By contrast, bronchoscopy appears to have greater value for early recognition (5, 7). Reported cases involving the central airway have described common bronchoscopic findings including white or grayish necrotic material, pseudomembranous covering, pale or necrotic mucosa, hyperemia and edema, and varying degrees of intraluminal obstruction or fixed stenosis (6–8, 11). Our patient exhibited several of these characteristic features and, despite successful control of the infection, ultimately developed glottic closure and severe subglottic stenosis. This course indicates that in patients with poor glycemic control, especially those with DKA, who present with necrotizing central airway lesions on bronchoscopy, the clinical focus should not be limited to determining whether a fungal infection is present. Instead, clinicians should promptly recognize the risk of vascular invasion and deep tissue destruction caused by mucormycosis so that deep specimen acquisition, histopathological confirmation, and airway risk stratification can be completed as early as possible (1, 5, 9, 10).

On the basis of early recognition and definitive etiologic diagnosis, systemic antifungal therapy remains the cornerstone of management for improving outcomes in ITM (1, 2, 12). International guidelines recommend liposomal amphotericin B as the preferred first-line treatment for mucormycosis and emphasize that therapy should be initiated as early as possible and at an adequate dose. Intravenous isavuconazole and intravenous or delayed-release tablet posaconazole may be considered first-line alternatives or salvage options in selected clinical settings (1, 2). Once the patient is clinically stable, step-down oral maintenance therapy with posaconazole or isavuconazole may be used, with treatment duration individualized according to radiologic response, disease extent, host immune and metabolic status, and control of the underlying disease (1, 2, 12). Previous studies have shown that delayed initiation of amphotericin B is associated with increased mortality (13). Published case reports and case-based reviews of central airway mucormycosis likewise suggest that more favorable outcomes are generally observed in patients who receive early systemic polyene therapy combined with aggressive airway management, whereas delayed diagnosis or insufficient treatment is more likely to result in persistent airway obstruction, respiratory failure, or death (5, 6). During treatment, an appropriate balance between efficacy and tolerability is essential, with close monitoring of hepatic and renal function, electrolyte disturbances, and potential drug–drug interactions according to the antifungal agent used (1, 12). For posaconazole, therapeutic drug monitoring may be considered, particularly when oral absorption is uncertain, clinical response is suboptimal, or there is a substantial risk of drug interactions (12). In addition, active control of the underlying disease together with metabolic and nutritional support is an important component of overall management.

In addition to adequate systemic antifungal therapy, necrotic tissue should be removed as early as feasible. For localized and resectable lesions, surgical debridement or resection remains one of the key measures associated with improved outcomes in mucormycosis (1, 12). In patients with central airway involvement, preservation of airway patency is equally critical. Previous case reports, small case series, and recent retrospective cohort studies suggest that bronchoscopic intervention can serve as an important component of multidisciplinary management, particularly for removal of necrotic tissue, local treatment, and relief of airway stenosis or obstruction (5–7, 14). However, long-term airway management strategies in these patients, especially in those requiring endotracheal intubation or tracheostomy support, remain poorly defined because current evidence is largely derived from individual case reports and retrospective single-center experience (5, 8, 14). Taken together with the previously reported cases summarized in Table 1, ITM can present with a wide spectrum of central airway involvement, ranging from subacute tumor-like tracheobronchial stenosis to rapidly progressive airway obstruction requiring emergent airway support (15–21).

**Table 1:** Summary of reported cases of invasive tracheal mucormycosis with major central airway involvement.

Study (year)	Patient (risk factors)	Airway presentation	CT	Bronchoscopy	Diagnosis	Airway intervention	Antifungal therapy	Key airway issues	Tracheostomy status	Outcome
Chaddha (2017) Ann Am Thorac Soc (15)	55 M; newly diagnosed diabetes mellitus	Progressive dyspnea; emergent endotracheal intubation required	Circumferential tracheal wall thickening (about 3 cm segment)	Necrosis of anterior and lateral tracheal walls	Airway biopsy confirmed; PAS/GMS positive	Tracheostomy; serial bronchoscopies; no surgical resection because of extensive involvement	Amphotericin B plus micafungin; step-down oral isavuconazole for 6 months after discharge	Extensive necrosis not amenable to resection; staged endoscopic control	Yes; about 7 months	Good general condition reported
Tuna (2025) Emerg Med Case Rep (16)	67 M; chronic kidney disease and diabetes mellitus; immunosuppression	Failed intubation due to subglottic obstruction; emergent tracheostomy	Subglottic-to-upper tracheal mass	Lumen filled with necrotic material	Tracheal biopsy pathology confirmed	Emergent tracheostomy; multiple endoscopic debridements	Liposomal amphotericin B with dose escalation (1 to 5 mg/kg/day)	Recurrent necrotic obstruction; delayed pathologic confirmation	Yes; about 20 days	Died on day 20 because of recurrent obstruction and hypercapnic respiratory failure
Deepak (2025) Lung India (17)	29 M; uncontrolled diabetes mellitus with diabetic ketoacidosis	Stridor; near-complete mid-tracheal obstruction	Intraluminal lesion	Friable necrotic airway segment (about 8 cm)	Tracheal biopsy confirmed; PAS/GMS positive	Rigid bronchoscopic debulking; silicone stent (15 × 80 mm) placed and later adjusted	Liposomal amphotericin B induction followed by posaconazole maintenance	Long-segment necrosis with loss of structural support; stent migration; restenosis	No; stent dependent	Long-term stent-dependent airway patency; imaging stable
Zheng (2025) Med Mycol Case Rep (18)	24F; poorly controlled type 1 diabetes mellitus	Worsened after tracheostomy; dyspnea; neck abscess	Peritracheal abscess with cavitation on neck CT	Disrupted tracheal cartilage rings	Histopathology plus molecular evidence supported Mucorales infection	Two surgical debridements and drainage; tracheostomy revision; ongoing airway and wound care	Liposomal amphotericin B plus nebulized amphotericin B, followed by isavuconazole	Tracheal rupture and extensive necrosis; tracheostomy-site complication	Yes; long term (not decannulated)	Radiologic improvement; outpatient treatment ongoing
Ramanan (2023) Acta Scientific Otolaryngology (19)	32F; newly diagnosed uncontrolled diabetes mellitus	Glottic stenosis with subglottic edema; emergent tracheostomy	Supraglottic and glottic edema on contrast-enhanced CT	Subglottic necrotic crust	Laryngeal and tracheal biopsy: PAS/GMS-positive mucor-like hyphae	Emergent tracheostomy; staged airway debridement; rehabilitation	Liposomal amphotericin B followed by posaconazole	Acute laryngo-subglottic obstruction; repeated debridement required	Yes; duration not reported	Decannulated
Damaraju (2024) Lung India (20)	49 M; uncontrolled diabetes mellitus	Life-threatening central airway obstruction; endotracheal intubation	Polypoid lesion on lateral tracheal wall	Yellow-gray necrotic tissue involving the carina	Lesion biopsy confirmed mucormycosis; PAS/GMS positive	Rigid bronchoscopic debulking plus electrocautery; covered metallic Y-stent, later removed	Liposomal amphotericin B followed by posaconazole maintenance	Immediate airway patency required before antifungals took effect	No; stent removed after about 2 months	Good outcome at 1-year follow-up
Kaliya (2024) Lung India (21)	69 M; poorly controlled type 2 diabetes mellitus; recent COVID-19; tobacco chewing and occasional smoking	Cough, fever, and breathlessness; tracheal stenosis initially mimicking malignancy	Distal tracheal and proximal main bronchial wall thickening with mucosal irregularity; PET/CT hypermetabolism	Distal tracheal narrowing with an endotracheal mass, nodularity, and proximal main bronchial lesions	Repeat bronchoscopic biopsies confirmed mucormycosis; GMS-positive broad, pauci-septate hyphae	No surgical intervention required	Liposomal amphotericin B 300 mg/day, 5 mg/kg/day, for 21 days plus posaconazole 300 mg/day	Tumor-like tracheobronchial stenosis; repeated biopsies required	No tracheostomy reported	Symptoms improved; bronchoscopy normalized after therapy; asymptomatic at 15-month follow-up
Present case	51F; poorly controlled type 2 diabetes mellitus with diabetic ketosis	Tracheostomy dependent; subglottic and tracheal obstruction by necrotic debris; recurrent hypoxemia	Pneumomediastinum and subcutaneous emphysema	Extensive white necrosis; suspected cartilage ring destruction	Broad, ribbon-like, pauci-septate hyphae; PAS/GMS positive; consistent with Mucorales	Multiple bronchoscopies; laser recanalization; ongoing tracheostomy care	Amphotericin B cholesteryl sulfate complex up to about 250 mg/day plus nebulized amphotericin B; step-down oral isavuconazole for about 4 months	Tenacious necrotic casts; cartilage destruction; high risk of decannulation failure	Yes; long term (not decannulated)	Persistent glottic closure and severe subglottic/tracheal stenosis

CT, computed tomography; PAS, periodic acid-Schiff; GMS, Grocott methenamine silver; Mucorales, the order of fungi that causes mucormycosis.

Most reported patients had identifiable predisposing factors, among which poorly controlled diabetes was the most common, sometimes in combination with DKA, chronic kidney disease, recent COVID-19, or immunosuppression (15–21). In terms of airway management, the priority during the acute phase is to restore and maintain effective ventilation while removing necrotic tissue under strict airway protection when needed. For patients with long-segment involvement, a high necrotic burden, damage to airway-supporting structures, or a high risk of recurrent obstruction, staged endoscopic intervention, tracheostomy, or stent placement may be required as part of supportive management (15–18, 20). However, the case reported by Kaliya et al. (21) also indicates that selected patients with localized tumor-like tracheobronchial involvement may improve with antifungal therapy and glycemic control without surgical airway intervention. Reported outcomes are heterogeneous, ranging from symptomatic and bronchoscopic resolution to decannulation or stent removal, long-term artificial airway or stent dependence, and death due to recurrent obstruction (15–21). Therefore, the therapeutic goal in ITM should not be limited to infection control alone, but should also include maintenance of airway patency and preservation of structural and functional integrity. On this basis, decannulation assessment in patients with ITM should be more cautious than in routine tracheostomy patients, with safety as the primary consideration. Before decannulation is attempted, clinicians should confirm that infection has been controlled and that there is no ongoing necrosis, while also comprehensively evaluating laryngotracheal structure, dynamic airway patency, vocal cord mobility, swallowing function, and cough effectiveness for secretion clearance. In the present case, long-term tracheostomy dependence was primarily attributable to persistent glottic closure and severe circumferential stenosis involving the subglottic region and trachea, highlighting the need for longitudinal prognostic assessment that balances airway safety, survival, and quality of life.

## 4 Conclusion

ITM progresses rapidly in high-risk hosts and carries a substantial risk of mortality. Early recognition depends on bronchoscopic evaluation and prompt etiologic confirmation. Management requires the simultaneous implementation of adequate antifungal therapy, correction of metabolic disturbances, and timely airway intervention. Even after infection control is achieved, severe structural airway damage may persist, indicating that clinical management should extend beyond infection control alone to include airway function preservation and long-term prognostic assessment.

## Data Availability

The original contributions presented in the study are included in the article; further inquiries can be directed to the corresponding author.
